# Effluent Osteopontin levels reflect the peritoneal solute transport rate

**DOI:** 10.1515/med-2021-0302

**Published:** 2021-06-07

**Authors:** Jianzhong Li, Jingjing Lan, Qing Qiao, Lei Shen, Guoyuan Lu

**Affiliations:** Department of Nephrology, The First Affiliated Hospital of Soochow University, Suzhou, Jiangsu 215006, People’s Republic of China

**Keywords:** peritoneal dialysis, Osteopontin, peritoneal solute transport rate

## Abstract

Long-term peritoneal dialysis (PD) is accompanied by low-grade intraperitoneal inflammation and may eventually lead to peritoneal membrane injury with a high solute transport rate and ultrafiltration failure. Osteopontin (OPN) is highly expressed through the stimulation of pro-inflammatory cytokines in many cell types. This study aimed to investigate the potential of OPN as a new indicator of peritoneal deterioration. One hundred nine continuous ambulatory PD patients were analyzed. The levels of OPN and IL-6 in peritoneal effluents or serum were analyzed by ELISA kits. The mean effluent OPN concentration was 2.39 ± 1.87 ng/mL. The OPN levels in drained dialysate were correlated with D/P Cr (*p* < 0.0001, *R* = 0.54) and D/D0 glucose (*p* < 0.0001, *R* = 0.39). Logistic regression analysis showed that the OPN levels in peritoneal effluents were an independent predictive factor for the increased peritoneal solute transport rate (PSTR) obtained by the peritoneal equilibration test (*p* < 0.001). The area under the receiver operating characteristic curve of OPN was 0.84 (95% CI: 0.75–0.92) in predicting the increased PSTR with a sensitivity of 86% and a specificity of 67%. The joint utilization of effluent OPN with age, effluent IL-6, and serum albumin further increased the specificity (81%). Thus, OPN may be a useful indicator of peritoneal deterioration in patients with PD.

## 1 Introduction

Peritoneal dialysis (PD) is a vital replacement therapy for patients with end-stage kidney disease. Approximately 11% of the population undergoing dialysis uses PD worldwide [1,2]. During PD, the peritoneal membrane (PM) naturally removes waste products and excess fluid from the blood and transports them to the dialysis solution. However, long-term exposure to hyperglycemic, hyperosmotic, and acidic dialysis solutions often causes low-grade chronic inflammation and PM injury. The PM presents as progressive fibrosis, angiogenesis, and vasculopathy, which lead to the increased solute transport and ultrafiltration failure [3,4,5,6]. Peritoneal equilibration test (PET) is widely used to assess the PM transport function and includes three parameters, namely, D/P Cr, D/D0 glucose, and 4 h ultrafiltration volume. In the PET results, the faster solute transfer rate is closely related to higher mortality and hospitalization rate [7]. However, PET requires multiple collection of blood and peritoneal effluents, and patients need to be hospitalized. In this regard, detecting relevant effluent-based biomarkers that can reflect the solute transfer rate is a more convenient detection strategy. Previous studies showed that effluent concentrations of interleukin 6 (IL-6), cancer antigen125 (CA125), vascular endothelial growth factor (VEGF), plasminogen activator inhibitor-1 (PAI-1), tenascin-C, and MMP families in dialysate obtained using PET were correlated with the D/P Cr ratio [8,9,10,11,12].

Osteopontin (OPN) is a highly phosphorylated glycophosphoprotein that can be secreted from many cell types, including epithelial cells, macrophages, osteoclasts, and fibroblasts in different tissues under inflammatory milieu. OPN participates in diverse important biological functions including inflammation, biomineralization, cell viability, tissue epithelial–mesenchymal transition (EMT), and fibrosis [13,14,15,16,17,18]. Moreover, OPN is localized in peritoneum tissues in patients with CAPD and is highly expressed accompanied with calcification deposits in inflammatory cells and fibroblast‐like cells in PM [19].

We suggest that OPN may be highly expressed and secreted from the PM through long-term and low-grade chronic inflammation stimulation during PD treatment. We investigated whether OPN may be a new potential indicator of PM injury characterized by high solute transport by analyzing the association between OPN levels in drained dialysate and peritoneal solute transport rate (PSTR) obtained with PET.

## 2 Patients and methods

### 2.1 Patients

From February 2018 to December 2018, 215 PD patients with end-stage renal disease at the PD unit of the First Hospital Affiliated of Soochow University were followed up regularly. We selected patients who were undergoing a CAPD prescription (four times exchanges per day). Other exclusion criteria included acute inflammatory processes, diagnosis of peritonitis or abdominal trauma within the past 6 months before the study, active autoimmune diseases, and tumors, with incomplete clinical characteristics. Eventually, 109 patients who were undergoing CAPD were analyzed. Table 1 summarized the clinical characteristics of the selected patients. All the patients in this study were dialyzed with the glucose-based PD fluid (Dianeal^®^, Baxter).

**Table 1:** Clinical characteristics of CAPD patients without peritonitis (*N* = 109). Clinical values are expressed as mean ± SD

Variable	Value
Gender (male/female)	57/52
Age (years)	49.14 ± 13.25
PD duration (months)	37.32 ± 35.01
D/P Cr	0.69 ± 0.11
D/D0 glucose	0.36 ± 0.07
4 h ultrafiltration volume (mL)	270.6 ± 138.4
Total Kt/V urea	1.82 ± 0.40
Peritoneal Kt/V urea	1.47 ± 0.35
Renal Kt/V urea	0.35 ± 0.44
Cholesterol (mmol/L)	4.31 ± 1.08
Triglyceride (mmol/L)	1.78 ± 1.33
Uric acid (μmol/L)	393.1 ± 89.18
Creatinine (μmol/L)	960.3 ± 292.8
Serum albumin (g/L)	34.39 ± 4.91
Hemoglobin (g/L)	96.61 ± 16.60
Serum calcium (mmol/L)	2.19 ± 0.58
Serum phosphate (mmol/L)	1.61 ± 0.49
PTH (pg/mL)	419.9 ± 27.78

Abbreviations: PD: peritoneal dialysis; D/P Cr: dialysate/plasma ratio of creatinine; D/D0 glucose: 4 to 0 h dialysate glucose; OPN: Osteopontin; PTH: parathyroid hormone.


**Ethics approval and consent to participate:** This study was performed with the written informed consent of all patients, and the procedure was approved by the Ethics Committee of The First Affiliated Hospital Soochow University.

### 2.2 PET

Semiquantitative assessment of the PM transport function was assessed with PET. Overnight (12 h) intraabdominal fluid was drained, and the PD fluid containing 2.5% dextrose solution was injected intraperitoneally. Four-hour dialysate creatinine concentration (D) was divided by plasma creatinine (P) concentration to obtain D/P Cr. Four-hour dialysate glucose concentration (D) was also divided by that obtained immediately after the injection (D0) to obtain D/D0 glucose. Ultrafiltration volume was measured. The grouping standard of the PET results was as follows: high transport (D/P Cr = 0.82–1.03), high–average transport (D/P Cr = 0.65–0.81), low–average transport (D/P Cr = 0.50–0.64), and low transport (D/P Cr = 0.34–0.49).

### 2.3 ELISA of OPN and IL-6

All participants in the experiment were asked to perform dialysis exchange according to the usual overnight dialysis regimen before visiting the PD center. Overnight effluent was fully drained the next morning in the PD center. We collected a 10 mL effluent sample and a 10 mL blood sample from each patient. The samples were stored at −80℃ immediately. The frozen blood samples were thawed on ice and centrifuged for 10 min at 1,500×*g*. The frozen peritoneal effluent samples were thawed on ice and centrifuged for 15 min at 2,500×*g*. OPN and IL-6 levels in peritoneal effluents or serum were quantified with the ELISA kit (CUSABIO China) according to the manufacturer’s instructions.

### 2.4 Statistical analysis

Clinical data were presented as means ± standard deviation (SD). Baseline characteristics of the study population were compared using Student’s-test or Mann–Whitney *U* test for normally or nonnormally distributed variables. Relationships between clinical variable and D/P Cr level were analyzed with Spearman’s correlation coefficient test. Independent factors affecting PSTR were analyzed by the logistic regression analysis. In addition, a logistic regression model was constructed for the probability of increased PSTR to identify the possible predictors of increased PSTR. The equation is as follows: probability = exp(*c*)/[1 + exp(*c*)], where *c* is 0.875 × effluent OPN levels + 0.06 × age – 0.139 × blood albumin + 0.037 × effluent IL-6 levels – 1.059. The optimal cutoff point was identified based on the maximum Youden index (sensitivity + specificity – 1). *p* < 0.05 (two tailed) was considered statistically significant. Statistical analyses were conducted using SPSS version 25.0 software (IBM Corp., USA).

## 3 Results

### 3.1 Patient characteristics

A total of 109 CAPD patients were enrolled in this study. Among these patients, 55.3% were males with a mean age of 49.14 ± 13.25 years and a median PD duration of 37.32 ± 35.01 months. OPN could be detected in the overnight peritoneal effluents with a mean concentration of 2.39 ± 1.87 ng/mL. Table 1 presents the clinical characteristics of the study.

### 3.2 Correlation between systemic and dialysate OPN and peritoneal transport characteristics

**Figure 1:**
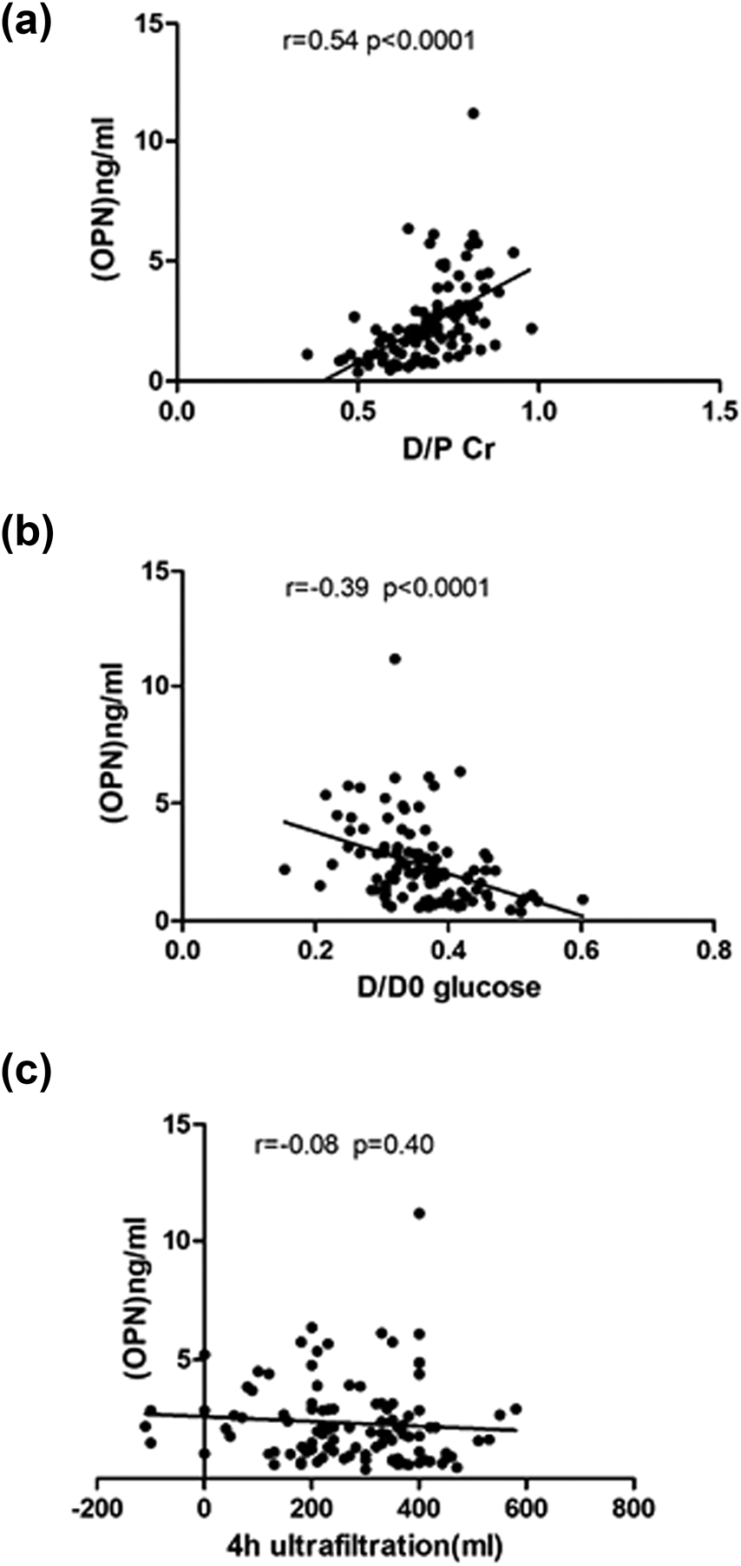
Correlation between OPN level in the peritoneal effluents and peritoneal transport characteristics. Peritoneal solute transport was assessed with the PET, and the levels of OPN in the overnight peritoneal effluents were also analyzed with ELISA. (a) The D/P Cr ratio versus the OPN level. (b) The D/D0 glucose ratio versus the OPN levels. (c) The 4 h ultrafiltration volume versus the OPN levels. D/P Cr, dialysate/plasma ratio of creatinine; D/D0 glucose, 4 to 0 h dialysate glucose; OPN, osteopontin.

The OPN concentration in overnight peritoneal effluents was correlated with PSTR determined using PET. OPN levels were correlated with D/P Cr (*p* < 0.0001, *r* = 0.54) and D/D0 glucose (*p* < 0.0001, *r* = −0.39) as determined through Spearman’s correlation coefficient test (Figure 1). In the blood samples, no firm correlation was observed between OPN levels and PET results (Table 2).

**Table 2:** Correlation between OPN level in serum and peritoneal transport characteristics. Peritoneal solute transport was assessed with the PET, the levels of OPN in serum were analyzed with ELISA

		4 h D/P Cr	D/D0 glucose	4 h ultrafiltration volume
Serum OPN	*r*	0.02	−0.13	−0.16
	*p*	0.77	0.65	0.74

Abbreviations: D/P Cr: dialysate/plasma ratio of creatinine; D/D0 glucose: 4 to 0 h dialysate glucose; OPN: Osteopontin.

### 3.3 Independent factors associated with peritoneal transport characteristics

**Figure 2:**
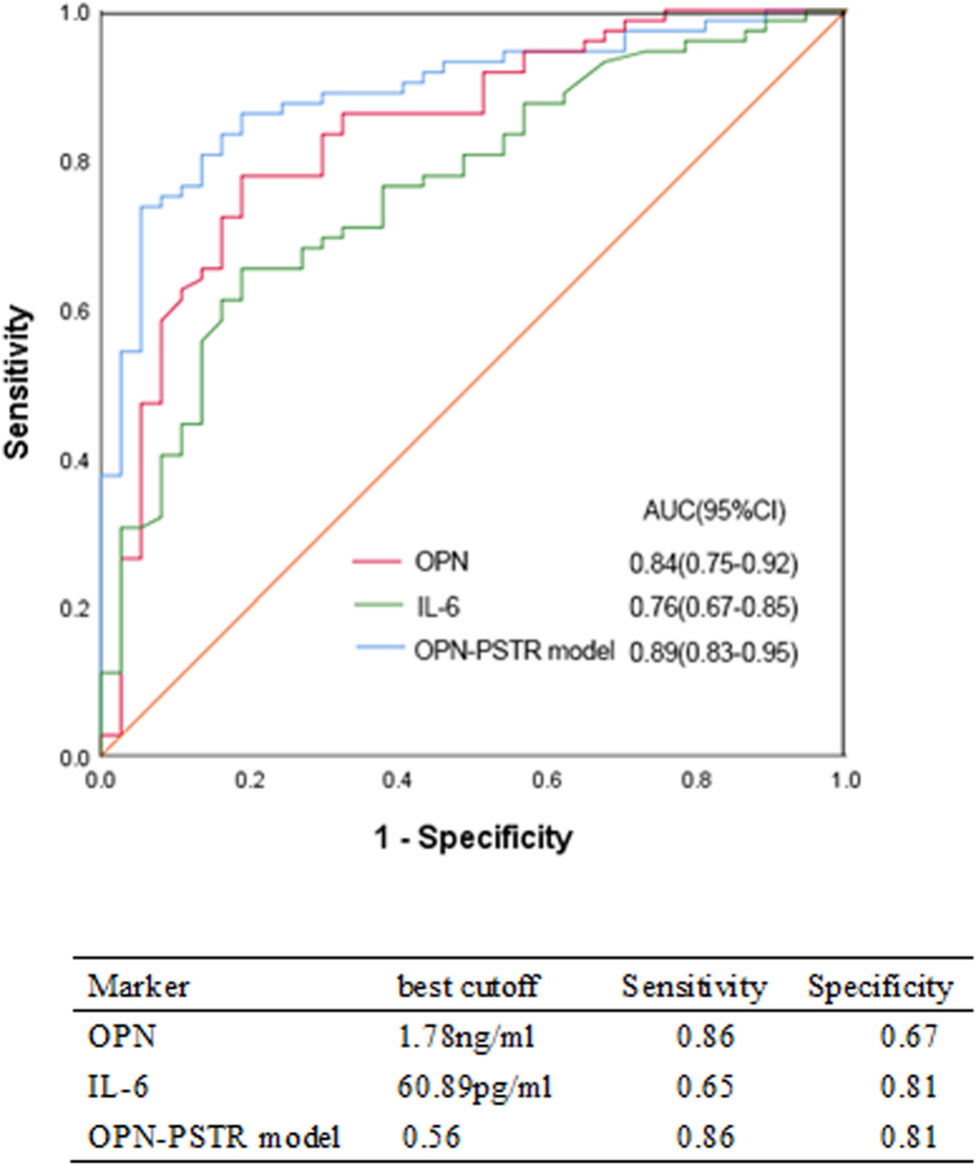
AUROC analyses of the predictors for identifying increased PSTR. OPN: Osteopontin; PSTR, peritoneal solute transport rate.

The patients were divided into either low and low–average transport (L/LA) group or high and high–average transport (H/HA) group based on the PET results. The OPN concentration in peritoneal effluents was considerably higher in the H/HA group than in the L/LA group (3.05 ± 1.94 vs 1.25 ± 1.03 ng/mL, *p* < 0.0001). The H/HA group was more likely to be older (51.57 ± 13.14 vs 44.41 ± 12.31, *p* = 0.007) and had lower serum albumin content (36.80 ± 3.17 vs 33.16 ± 5.19 g/L, *p* < 0.0001) and lower serum phosphate (1.51 ± 0.41 vs 1.79 ± 0.58 mmol/L, *p* = 0.004) and effluent IL-6 levels (45.8 ± 22.04 vs 74.13 ± 35.51 pg/mL; Table 3). Logistic regression was employed to identify independent predictive factors for increased PSTR (H/HA). OPN or IL-6 in effluents, age, and serum albumin were independent predictive factors for the increased PSTR, whereas PD duration, gender, and serum phosphate were not (Table 4). The diagnostic accuracy of the markers in effluents measured for identifying the increased PSTR was examined. Relative analysis showed that the area under the receiver operating characteristic (ROC) curve of IL-6 was 0.76 (confidence interval (CI): 0.67–0.85, *p* < 0.001) in predicting increased PSTR with a sensitivity of 65% and a specificity of 81%. The ROC curve of OPN was 0.84 (95% CI: 0.75–0.92, *p* < 0.001) in predicting increased PSTR with a sensitivity of 86% and a specificity of 67%. The OPN-PSTR model was constructed by using four variables (age, serum albumin, effluent OPN, and effluent IL-6). The model showed increased specificity (81%; Figure 2).

**Table 3:** Data are presented as means ± standard deviation (SD) or *n* (%)

Variable	L/LA (*n* = 37)	H/HA (*n* = 72)	*p*
Male gender (%)	0.54	0.51	0.794
**Serum albumin (g/L)**	36.80 ± 3.17	33.16 ± 5.19	**<0.0001**
Hemoglobin (g/L)	100.47 ± 18.62	94.62 ± 15.21	0.081
PD duration (months)	30.09 ± 24.50	41.03 ± 38.96	0.123
**Age (years)**	44.41 ± 12.31	51.57 ± 13.14	**0.007**
**Serum phosphate (mmol/L)**	1.79 ± 0.58	1.51 ± 0.41	**0.004**
Serum calcium (mmol/L)	2.25 ± 0.29	2.16 ± 0.67	0.454
Triglyceride (mmol/L)	2.08 ± 1.37	1.62 ± 1.29	0.084
Cholesterol (mmol/L)	4.37 ± 0.99	4.45 ± 1.17	0.715
Uric acid (μmol/L)	391.31 ± 82.40	394.07 ± 92.02	0.879
**D/P Cr**	0.57 ± 0.06	0.75 ± 0.07	**<0.0001**
**D/D0 glucose**	0.43 ± 0.06	0.33 ± 0.05	**<0.0001**
**Ultrafiltration volume (mL)**	316.22 ± 121.40	247.21 ± 141.45	**0.01**
Creatinine (μmol/L)	990.78 ± 333.16	944 ± 270.94	0.438
PTH (pg/mL)	470.72 ± 260.12	394.44 ± 294.68	0.197
Total Kt/V	1.85 ± 0.42	1.79 ± 0. 39	0.373
Peritoneal Kt/V	1.44 ± 0.32	1.49 ± 0.37	0.440
Renal Kt/V urea	0.43 ± 0.50	0.32 ± 0.40	0.174
Serum OPN (ng/mL)	32.45 ± 13.03	37.82 ± 17.31	0.771
Serum IL-6 (pg/mL)	29.33 ± 20.11	33.98 ± 17.77	0.892
**Effluent OPN (ng/mL)**	1.25 ± 1.03	3.05 ± 1.94	**<0.0001**
**Effluent IL-6 (pg/mL)**	45.80 ± 22.04	74.13 ± 35.51	**<0.0001**

Abbreviations: PD: peritoneal dialysis; D/P Cr: dialysate/plasma ratio of creatinine; D/D0 glucose: 4 to 0 h dialysate glucose; OPN: Osteopontin; PTH: parathyroid hormone. Bold letters and values indicate statistically significant difference.

**Table 4:** A logistic regression model for the predictors of PSTR in PD patients, adjustment for age, gender, PD duration, serum albumin, serum phosphate, effluent OPN, and effluent IL-6

	*B*	Wald	*p* value	OR	95% CI	
					Lower	Upper
Age	0.060	5.431	0.020	1.062	1.010	1.116
Albumin	−0.139	4.406	0.036	0.870	0.764	0.991
OPN	0.875	9.345	0.002	2.400	1.369	4.206
IL-6	0.037	9.062	0.003	1.038	1.013	1.063
Constant	−1.059	0.139	0.709	0.347		

Abbreviation: OPN: Osteopontin.

## 4 Discussion

### 4.1 Principal findings and comparison with other studies

This study showed correlations between OPN levels in dialysate and D/P Cr or D/D0 glucose, and the OPN level in effluents was an independent predictive factor for the increased PSTR. However, we did not observe obvious relevance between OPN levels in effluents and 4 h ultrafiltration volume. On the basis of the results of a large retrospective study that enrolled more than 10,000 patients with PD subjected to PET, a strong inverse association was found between D/P Cr and D/D0 glucose (*r* = 0.84), and a linear association existed between these parameters and the hospitalization rate or all-cause mortality [7,20]. However, only a modest correlation was observed between 4-hour ultrafiltration volume and D/P creatinine or D/D0 glucose, and in addition, no correlation existed between 4-hour ultrafiltration volume and all-cause mortality [7]. Thus, D/P Cr and D/D0 glucose are superior to 4-hour ultrafiltration volume in identifying the increased PSTR at risk of adverse clinical outcomes. The lower predictive value of 4 h ultrafiltration volume maybe due to the following reasons: (1) except PM transport function, the variable residual renal function of CAPD patients is also an essential determinant of the 4 h ultrafiltration volume. (2) Since commercially available PD bags contain more dialysate volume than prefilling flushing, this is variable, resulting in inaccurate measurement [21]. Taken together, effluent OPN may be an excellent predictor for PET results and prognosis of patients with PD.

In this study, 66% of the patients followed up in our center were categorized into the H/HA group based on the PET results; this finding is similar to those reported by other multicenter studies (47.4–83%) [22,23,24]. The comparison of H/HA and L/LA groups showed statistically significant differences between serum albumin and serum phosphate levels. Various studies reported that high peritoneal transport status is accompanied by high baseline albumin clearance [7,23,25,26]. Moreover, patients with fast transport present with severe systemic inflammation status, and pro-inflammatory cytokines could cause muscle wasting and suppress appetite [27,28]. Thus, patients with CAPD in the H/HA group had lower serum albumin compared with those in the L/LA group. Phosphate transport across the peritoneum is regulated by osmotic, chemical, and electrical gradients as well as by transmembranous active phosphate transporters; thus, this process is complex. Bernardo et al. reported that phosphate clearance increased in fast transporters compared with that in slow transporters in patients with CAPD and APD [29]; this finding is consistent with our results.

Increasing lines of evidence show that chronic inflammation might be an initial factor for PM injury. After prolonged PD treatment, MCs and peritoneal macrophages produce a wide array of inflammatory cytokines, such as IL-1β, tumor necrosis factor-α (TNF-α), and IL-6, eventually leading to PM damage and functional abnormalities [5,27,30]. The secretion of OPN is enhanced in the inflammatory microenvironment, whereas TNFα and IL-1β stimulate the transcription and the release of OPN genes [13]. Consistent with the previous studies, IL-6 levels in effluents were higher in the H/HA group than that in the L/LA group and could be an independent predictive factor for increased PSTR. No extreme change in the serum OPN levels was found as the PSTR increased. Overall, the high OPN concentration in the H/HA group may be attributed to the high number of pro-inflammatory cytokines released from the PM. The cellular source of PM, where OPN is synthesized and released, merits further investigation.

### 4.2 Limitations of study

This study features several important limitations. First, the cross-sectional nature of the study excluded the establishment of causal relationships and temporal trends between OPN levels in effluents and PSTR. Second, we did not investigate the association between OPN levels in effluents and adverse outcomes in PD. Third, this single-center study involved a small sample size, and selection bias might be present considering that a large number of subjects were excluded due to the strict inclusion and exclusion criteria.

### 4.3 Summary

In conclusion, we established the associations between OPN levels in the drained dialysate and D/P Cr and D/D0 glucose. Effluent OPN is also an independent predictive factor for the increased PSTR. The OPN-PSTR model, constructed by using four variables (age, serum albumin, effluent OPN, and effluent IL-6) demonstrated good diagnostic performance for accurately identifying increased PSTR.

## Abbreviations

PD: peritoneal dialysis
OPN: Osteopontin
CAPD: continuous ambulatory PD
PET: peritoneal equilibration test
PSTR: peritoneal solute transport rate
ROC: receiver operating characteristic
PM: peritoneal membrane
EMT: epithelial–mesenchymal transition
IL-6: interleukin 6
CA125: cancer antigen 125
VEGF: vascular endothelial growth factor
PAI-1: plasminogen activator inhibitor-1
MMP: matrix metalloproteinase
D/P Cr: dialysate/plasma ratio of creatinine
D/D0 glucose: 4-to 0-hour dialysate glucose
